# Soil-atmosphere fluxes of CO_2_, CH_4_, and N_2_O across an experimentally-grown, successional gradient of biocrust community types

**DOI:** 10.3389/fmicb.2022.979825

**Published:** 2022-09-26

**Authors:** Andrew D. Richardson, Gary V. Kong, Katrina M. Taylor, James M. Le Moine, Matthew A. Bowker, Jarrett J. Barber, David Basler, Mariah S. Carbone, Michaela Hayer, George W. Koch, Mark R. Salvatore, A. Wesley Sonnemaker, David E. Trilling

**Affiliations:** ^1^Center for Ecosystem Science and Society, Northern Arizona University, Flagstaff, AZ, United States; ^2^School of Informatics, Computing, and Cyber Systems, Northern Arizona University, Flagstaff, AZ, United States; ^3^Department of Astronomy and Planetary Science, Northern Arizona University, Flagstaff, AZ, United States; ^4^University of California, Santa Barbara, CA, United States; ^5^Department of Astronomy and Astrophysics, The Pennsylvania State University, State College, PA, United States; ^6^School of Forestry, Northern Arizona University, Flagstaff, AZ, United States; ^7^Department of Environmental Sciences–Botany, University of Basel, Basel, Switzerland; ^8^Swiss Federal Institute for Forest, Snow and Landscape Research WSL, Birmensdorf, Switzerland; ^9^Lowell Observatory, Flagstaff, AZ, United States

**Keywords:** biological soil crusts, cryptobiotic crusts, cyanobacteria, greenhouse gas fluxes, nitrification, methane, photosynthesis, succession

## Abstract

Biological soil crusts (biocrusts) are critical components of dryland and other ecosystems worldwide, and are increasingly recognized as novel model ecosystems from which more general principles of ecology can be elucidated. Biocrusts are often diverse communities, comprised of both eukaryotic and prokaryotic organisms with a range of metabolic lifestyles that enable the fixation of atmospheric carbon and nitrogen. However, how the function of these biocrust communities varies with succession is incompletely characterized, especially in comparison to more familiar terrestrial ecosystem types such as forests. We conducted a greenhouse experiment to investigate how community composition and soil-atmosphere trace gas fluxes of CO_2_, CH_4_, and N_2_O varied from early-successional light cyanobacterial biocrusts to mid-successional dark cyanobacteria biocrusts and late-successional moss-lichen biocrusts and as biocrusts of each successional stage matured. Cover type richness increased as biocrusts developed, and richness was generally highest in the late-successional moss-lichen biocrusts. Microbial community composition varied in relation to successional stage, but microbial diversity did not differ significantly among stages. Net photosynthetic uptake of CO_2_ by each biocrust type also increased as biocrusts developed but tended to be moderately greater (by up to ≈25%) for the mid-successional dark cyanobacteria biocrusts than the light cyanobacterial biocrusts or the moss-lichen biocrusts. Rates of soil C accumulation were highest for the dark cyanobacteria biocrusts and light cyanobacteria biocrusts, and lowest for the moss-lichen biocrusts and bare soil controls. Biocrust CH_4_ and N_2_O fluxes were not consistently distinguishable from the same fluxes measured from bare soil controls; the measured rates were also substantially lower than have been reported in previous biocrust studies. Our experiment, which uniquely used greenhouse-grown biocrusts to manipulate community composition and accelerate biocrust development, shows how biocrust function varies along a dynamic gradient of biocrust successional stages.

## Introduction

Biocrusts, or biological soil crusts—also referred to as microbiotic crusts, cryptobiotic crusts, or cryptogamic crusts—cover 12% of the earth’s land surface (Rodriguez-Caballero et al., 2018), and are especially prevalent in drylands where water availability is limiting for vascular plants (Belnap et al., 2016). Existing as a thin interface, just millimeters thick, between the soil surface and the surrounding environment, biocrusts have been frequently described as a “living skin” (Belnap et al., 2016; Bowker et al., 2018; Li et al., 2021) and also a “downsized critical zone” (Pointing and Belnap, 2012) that can serve as a unique model ecosystem (Bowker et al., 2014). Despite their inherently low productivity (Bowker et al., 2018), biocrusts are complex and diverse communities consisting of a matrix of soil particles together with both prokaryotic and eukaryotic organisms from a wide range of lineages and metabolic strategies.

Biocrusts provide numerous important ecosystem services and play a vital role in maintaining ecosystem health and stability (Elbert et al., 2012). The cyanobacteria, algae, lichens, mosses, and liverworts that comprise most biocrust communities are critical as photoautotrophic primary producers (Ferrenberg et al., 2017), while some cyanobacteria—both free living and in symbiotic association with mosses and lichens—fix atmospheric nitrogen (Barger et al., 2013). In this way, biocrusts supply C and N to support consumers and decomposers at the scales of communities to ecosystems. The amounts of both C and N fixed by biocrust communities are large enough to be significant in the context of global budgets (Belnap, 2001; Elbert et al., 2012; Barger et al., 2016; Weber et al., 2016a). At the soil surface, biocrusts aggregate and cement soil particles and reduce erosion, influence soil hydrology and water balance and the fate of precipitation, and alter the soil thermal regime through effects on albedo and energy balance (Belnap et al., 2013; Ferrenberg et al., 2017; Bowker et al., 2018; Rodriguez-Caballero et al., 2022). Thus, biocrusts are important regulators of the biotic and abiotic environment of dryland ecosystems, and also relevant at larger scales in the context of global biogeochemical cycling.

A global biocrust research community has only coalesced over the last 25 years (Belnap et al., 2016; Bowker et al., 2018), and it is therefore hardly surprising that, compared to other ecological systems, many aspects of biocrust development and function remain remarkably under-studied (Ferrenberg et al., 2017). For example, a century of research on forest ecosystems has led to well-developed theories of forest succession and stand dynamics, and ever-increasing understanding of how these drive productivity (Kira and Shidei, 1967; Odum, 1969; Ryan et al., 1997; Tang et al., 2014), stand structure (Oliver and Larson, 1996), and CO_2_ exchange (Barford et al., 2001; Goulden et al., 2011). For biocrusts, comprehensive theory of this nature is lacking (Ferrenberg et al., 2017). Although substantial progress has been made in the two decades since Belnap and Lange (2001) commented on the “very limited” state of knowledge regarding photosynthesis and CO_2_ exchange of biocrusts in different ecosystems, there are still only a few dozen papers on biocrust photosynthesis, compared to the thousands of publications on photosynthesis by vascular plants. More generally, understanding of the potential for biocrusts to serve as sources or sinks of greenhouse gases including CO_2_, CH_4_, and N_2_O is nowhere near as advanced as it is for forest, grassland, and agricultural ecosystems.

It is generally thought that biocrust successional patterns often, but not always, follow a fairly predictable trajectory, with cyanobacteria and algae being characteristic of early successional stages, and lichens and bryophytes representing late successional stages (Weber et al., 2016b). Adding to our understanding of biocrust successional pathways, exceptions to the general patterns—and what drives these exceptions—are beginning to be investigated (Lan et al., 2015; Read et al., 2016; Weber et al., 2016b). Yet, there remains a general lack of empirical data linking structure (e.g., community composition) and function (e.g., productivity and CO_2_ exchange, nitrogen cycling) over the whole trajectory of biocrust succession. While some previous studies have measured biocrust trace gas fluxes under different environmental conditions and for different biocrust types (Lange, 2001; Grote et al., 2010; Jia et al., 2018; Tamm et al., 2018; Lafuente et al., 2020), comparatively few studies have made gas flux measurements along a well-defined biocrust successional gradient, or over time following mechanical disturbance.

Therefore, the overall objective of this work was to investigate how trace gas fluxes of CO_2_, CH_4_, and N_2_O varied across a successional gradient of biocrust types, and over time as biocrusts of each type developed. We carried out a microcosm greenhouse experiment where, in addition to bare-soil controls, we grew (using locally-sourced inocula) three different types of biocrust communities to represent early-, mid-, and late-successional stages typical of biocrusts of the American Southwest. We periodically quantified the visible community composition of each biocrust, and each week we measured the net soil-atmosphere exchange of CO_2_ and CH_4_. At the end of the experiment, we conducted a final set of flux measurements that included N_2_O in addition to CO_2_ and CH_4_. To characterize microbial community composition and diversity, we performed amplicon sequencing of the bacterial and fungal communities of a subset of samples representing each biocrust type.

Based on our reading of the existing literature, our understanding of successional patterns in biocrusts and other ecosystem types, and our knowledge of biocrust ecology and biogeochemical processes, we made a set of predictions that we would test against data from our experiment. We predicted that net CO_2_ uptake would increase as a function of biocrust development (i.e., regaining of form and function from a disturbed state) and successional stage as photosynthetic biomass increased, with later successional stages taking up more CO_2_ than early successional stages. We predicted that CH_4_ flux would largely be controlled by environmental parameters such as soil moisture and temperature and would be weakly linked to biocrust development or successional stage. Finally, we predicted that N_2_O flux would be small but detectable, and that it would be positively associated with successional stages that have a greater prevalence of N-fixing organisms and greater N availability. Our experiment provides rare simultaneous measurements of multiple trace gas fluxes from biocrust communities, and demonstrates that biocrust function varies in space and time, and continuously in relation to biocrust successional development.

## Materials and methods

### Experimental design

We created microcosms containing actively growing biocrusts of different successional stages in the Northern Arizona University Research Greenhouse in Flagstaff, AZ, USA. Our study design created wide range of samples with differing degrees of biocrust development due to (1) initial inoculation with early-, mid- and late-successional types, (2) two staggered “sets” of samples, differing by about 2 months in age, and (3) continuous development over the duration of the experiment, as form and function recover from a low-biomass, disturbed state following inoculation.

Each microcosm was prepared in a 739 ml, square plastic container (25 fluid oz. GladWare brand plastic food storage containers; Glad Products Company, Oakland, CA, USA), with several 0.5 mm holes drilled through the bottom to allow entry of irrigation water. After lining the bottom of the container with a permeable barrier (generic weed control fabric) to reduce soil loss, we backfilled each experimental unit with 200 ml of sterilized dune sand sourced near Arches National Park, UT, USA. We chose this sand because of previous success growing many types of biocrusts over it (Doherty et al., 2015; Antoninka et al., 2016; Bowker et al., 2021), and because it was a reasonable analog for the soils from which our biocrust materials originated (sand properties are documented in Bowker and Antoninka, 2016).

Over the top of each sand-filled container, we dispersed 18 ml of field-collected inocula representing three different biocrust types: light cyanobacteria, dark cyanobacteria, and moss-lichen dominated biocrusts. In addition, we prepared uninoculated, bare soil controls featuring no addition of biocrust from the field. Our three biocrust types represent a successional gradient typical of drylands of the southwestern United States. Light cyanobacteria biocrusts are typically dominated by motile, sheathed filamentous cyanobacterial taxa and represent an early-successional state of development in our study area. Dark cyanobacteria biocrusts contain a substantial proportion of surface-bound, dark-pigmented, and often N-fixing cyanobacterial taxa in addition to the motile, bundled, filamentous taxa; these represent a mid- to late-successional state of development in our study area. Moss-lichen biocrusts are visually dominated by various species of mosses and or lichens, and also contain cyanobacterial taxa; these represent a later successional community in our study area.

All three types of biocrust inocula were collected in the field from a location in Sedona, AZ, USA. We identified areas colonized by each type of biocrust and sampled each area separately. We carefully lifted and removed the top 0.5–1 cm of soil using a narrow, flat trowel. For each type, many spatially discrete subsamples were collected within one ∼2 ha area, pooled and homogenized to create one composite sample of each type from which to inoculate our microcosms. For moss-lichen subsamples, we intentionally included a diversity of different moss and lichen taxa in our collections. For each biocrust type, the composite sample was passed through a 4 mm sieve to disaggregate it, and repeatedly stirred to homogenize it. We further purified the sieved moss-lichen material by allowing detached soil to then pass through a 2 mm sieve with gentle shaking, and retaining the biomass atop the soil. Through sieving, we were able to finely divide our inoculum material, and then mix it well. This then allowed us to evenly disperse well-mixed biocrust fragments across the surface of each sample, which resulted in experimental units that had representative subsamples of the inoculum and an even distribution of the inoculum across the surface. Thus sieving allowed us to minimize the within-treatment variation across replicate samples.

For each of our four treatments (bare soil controls plus three biocrust types), we established 10 replicate microcosms, each in its own separate plastic container. Furthermore, we had two different inoculation sets, which we tracked with the measurements described below. “Set 1” was inoculated on April 8, 2021, and “Set 2” was inoculated on June 3, 2021. Our objective with the two sets was to have both older and younger biocrusts that varied in age from days to months since inoculation. This gave us a total of 80 samples (4 treatments × 10 replicates × 2 sets).

After inoculation, the biocrust microcosms grew in an automatically irrigated system based on Doherty et al. (2015). Briefly, each plastic GladWare container was nested within another container of the same dimensions, each with a small diameter drainage hole. In scheduled pulses of 15 s in the morning and afternoon, our system delivered charcoal-filtered water to each lower basin, where it pooled, and subsequently entered the upper basin and rose through the sand *via* capillary action, hydrating the biocrust organisms. At the end of each pulse, water drained from both basins, leaving them at field capacity. This system operated 7 days a week and was sufficient to maintain a continuously hydrated state in growing biocrusts, with the exception of one planned drying event to control unwanted molds, and one unplanned event caused by a power outage. Containers were covered with 50% shade cloth both to prevent supraoptimal temperatures and to slow evaporation rates of water. The greenhouse was cooled using a thermostat-controlled evaporative cooler, maintaining daily mean (24 h μ ± 1 SD across days) temperatures at about 17.7 ± 5.6°C. Our gas exchange measurements were conducted during the day, when temperatures were warmer (24.6 ± 4.3°C, μ ± 1 SD across sample points). Incoming light was not controlled, but PPFD (photosynthetic photon flux density) inside the greenhouse is typically about half that outside. Relative humidity was not controlled, but is typically between 25 and 65%.

### Characterization of biocrust community composition

To document changes in the biocrust community, we measured percentage cover using a grid-point intercept method and a gridded quadrat (Belnap et al., 2006). At each monitoring event we recorded 25 intersections per microcosm. We differentiated mosses and lichens to the greatest taxonomic resolution possible, which was typically genus or species. We differentiated interceptions of light cyanobacterial communities and dark cyanobacterial communities from bare, uncolonized soil, and other surface covering elements (e.g., some rock fragments and pieces or vascular plant litter were incidentally introduced with the inocula). Additional biological elements were observed, especially later in the experiment, and we added cover categories to capture them (e.g., grass-green patches consistent with green algae, fungal fruiting bodies, etc.). In addition to recording 25 intersections per unit, we also noted any components that were present within the microcosm, but not intercepted and assigned them a value of 0.5 intersections (the halfway point between the detection limit of our method and 0). These values were converted to cover for each taxon/category by dividing the number of intersections of that type by the total number of intersections recorded in the unit. We also calculated the richness as the number of biological taxa/categories observed per unit. We monitored cover on May 3–4, 2021 (Set 1 only), and June 18, 2021 and August 13–17, 2021 (both Set 1 and Set 2).

### Measurements of CO_2_, CH_4_, and H_2_O fluxes

To characterize physiological activity of biocrust communities, we measured the net exchange of CO_2_ and CH_4_ of each biocrust microcosm on a weekly basis using a custom-built manual chamber system. The system (Supplementary Figure 1) consisted of a wooden base on which a GladWare plastic container (identical to the containers in which samples were grown) was mounted, a flat clear acrylic (240 mm × 240 mm, 5.6 mm thickness, Pokono Laser Cutting, Oakland, CA, USA) chamber lid with foam gasket that was connected with a hinge to a wooden block glued to the base, and an adjustable metal clamp (model 5127A12 Auto-Adjusting Hold-Down Toggle Clamp, McMaster-Carr, Atlanta, GA, USA). The chamber lid also contained gas inlet and outlet ports (model PP1208W 1/4OD Bulkheads, John Guest USA, Inc., Atlanta, GA, USA), a thermistor to measure chamber air temperature (model 22/420/3 Digital Refrigerator Thermometer, Brannon, Cleator Moor, Cumbria, England), a small fan for mixing air within the chamber (Raspberry Pi 4 Fan, iUNiKER Pi, Shenzhen, Guandong, China), and a 3 mm thick adhesive closed cell Neoprene gasket (Storystore, Guangzhou, Guandong, China). Measurements were made with the entire chamber system illuminated with supplemental lighting (model 189X-PRO LED Grow Light, Hydro Grow LED, Monroeville, PA, USA) to standardize, as much as possible, lighting conditions across measurements.

For each measurement, the sample container was nested within the chamber system’s GladWare container, a second 2-layer closed cell Neoprene gasket was placed on the container, and then the chamber lid was lowered on top of the sample container and sealed tightly with the clamp. Chamber air temperature and ambient light level (model LI-189 PPFD quantum sensor, LI-COR Biosciences, Lincoln, NE, USA) were recorded for each measurement. Air was circulated for 3 min from the chamber headspace through a OF-CEAS (Optical Feedback—Cavity Enhanced Absorption Spectroscopy) CO_2_/H_2_O/CH_4_ gas analyzer (model LI-7810, LI-COR Biosciences, Lincoln, NE, USA) and returned to the chamber. We recorded the change in CO_2_ and CH_4_ (reported as dry air concentrations, ppm and ppb, respectively) every 1 s. We fit a linear model using least squares regression to determine the rate of gas accumulation for each gas species. The slope of the linear model, the chamber air space volume (887 cm^3^), and the biocrust surface area (0.0212 m^2^) were used to calculate the flux rate (mol m^–2^ s^–1^) for each gas. Molar fluxes are based on concentrations adjusted for temperature and atmospheric pressure.

At the conclusion of the experiment, we conducted (August 17–19, 2021) a final set of flux measurements using the system and protocol described above, but with a second (N_2_O/H_2_O) gas analyzer (model LI-7820, LI-COR Biosciences) connected in parallel (i.e., the inlet and outlet tubes from the chamber were divided so half the flow went to each analyzer) to enable simultaneous quantification of net exchange of N_2_O. This analyzer had not been available for the weekly measurements conducted earlier in the summer, and thus this final set of measurements was largely motivated by the unique opportunity presented by our experiment to investigate biocrust N_2_O fluxes along a successional gradient. For this round of measurements, after each sample was measured under sunlit conditions the entire chamber system was covered with a light-blocking box and the flux measurements were repeated. The CO_2_ flux measured when the chamber was sunlit is interpreted to be net photosynthesis (i.e., net ecosystem exchange), the CO_2_ flux measured in the dark is interpreted to be ecosystem respiration (autotrophic plus heterotrophic), and their difference thus provides a measure of gross ecosystem photosynthesis.

During the weekly CO_2_/CH_4_ flux measurements, and the final CO_2_/CH_4_/N_2_O flux measurements, we routinely included empty GladWare containers (“blanks”) in the set of samples measured each day. This was done both for quality control and to quantify potential trace gas production from the plastic containers themselves (Royer et al., 2018).

Based on the manufacturer’s specifications, and the design of our chamber system, the minimum detectable flux (calculated from a chamber concentration change over 2 min equal to the analyzer’s reported precision) is estimated to be 0.023 μmol m^–2^ s^–1^ for CO_2_, 0.004 nmol m^–2^ s^–1^ for CH_4_, and 0.003 nmol m^–2^ s^–1^ for N_2_O.

### Statistical analysis of trace gas fluxes

#### Data cleaning and outlier detection

Data cleaning involved screening fluxes for outliers, and then visually inspecting the time series of concentration data from which the fluxes were calculated to evaluate the validity of those questionable measurements. For example, a handful of CH_4_ flux measurements (all of which indicated CH_4_ consumption) were discarded because either the time series suggested the measurement chamber was not sealed properly, or because the initial CH_4_ concentration was substantially elevated above ambient and “uptake” was observed as (presumably) CH_4_ diffused into the soil. Once outliers were discarded, residual variance across treatments was approximately constant, although for the CO_2_ flux measurements the residual variance for the control treatment was substantially smaller than the residual variance for any of the three biocrust treatments (our models, as described below, accounted for this when necessary). The discarded values are assumed to be missing completely at random, and should not bias the subsequent analysis.

#### Mixed model analysis of treatment effects

Statistical analysis consisted of an initial investigation using generalized additive models (GAM) to explore the structure of random effects, environmental factors (air temperature, soil moisture, and PPFD), and interactions of these covariates with treatment (biocrust type) and with each other. Using model selection criteria (likelihood ratio tests, AIC, BIC, and adjusted *R*^2^) and the principle of parsimony, we progressively simplified models by eliminating two-way interactions and replacing smoothing splines with quadratic terms, where justified. As our primary interest was to understand differences among biocrust types and how these changed with biocrust development (i.e., age), rather than characterize the response of different biocrusts to the environmental factors, we emphasize the main treatment effects below; other covariates in the model were essentially used to account for residual variance and reduce the standard errors on the main treatment effects, although these are discussed when they can provide insight into the underlying biology. Analyses were conducted using the *mgcv* (Wood, 2017) and *nlme* (Pinheiro et al., 2022) packages in R.

Because of differences in signal-to-noise ratio for different gas species (very high for CO_2_, but much lower for CH_4_ and N_2_O due to the much smaller fluxes of these gases) and differences in sample size between sets of measurements (very large for the weekly measurements of CO_2_ and CH_4_ flux, but much smaller for the end-of-summer measurements of CO_2_, CH_4_, and N_2_O flux), the complexity of the final model varied among individual analyses. For our analysis of the weekly CO_2_ flux measurements, we ended up with a mixed effects model which accounted for random intercepts, differences in residual variance among treatments, and interactions of age and environmental covariates with treatments. For our analysis of the weekly CH4 flux measurements, we ended up with a somewhat simpler regression (ANCOVA) model in which there were no interactions with the effects of treatment, age and light, and other environmental factors accounted for additional variability.

For our analysis of the end-of-summer measurements of CO_2_, CH_4_, and N_2_O, we ended up with even simpler ANCOVA models; factors considered for inclusion included treatment, biocrust age (in this case, a binary variable distinguishing between Set 1 and Set 2 biocrusts), as well as air temperature, soil moisture content, and PPFD. However, following our model selection procedure, only treatment, air temperature (CO_2_ and CH_4_ only), and PPFD (N_2_O only) were retained.

### Determination of soil water content and organic matter content

We anticipated that the water content of each sample could be an important covariate that would explain some of the variation in measured gas fluxes. Therefore, to enable determination of gravimetric water content (g H_2_O/g oven dry soil) of each sample on each flux measurement date, each sample container was weighed to the nearest 0.001 g (Adventurer Pro model AV53, Ohaus Corporation, Parsippany, NJ, USA) every time fluxes were measured, providing a “fresh” (i.e., wet) mass. After the conclusion of the experiment, samples were first air-dried for several weeks and then air-dried subsamples (ca. 140 g) were oven dried (model OV-560A-2, Blue M, Blue Island, IL, USA) at 60°C for at least 24 h, then re-weighed to give the dry mass. Actual wet and dry soil mass were then calculated by subtracting off the mass of the GladWare container (28.9 ± 0.1 g, μ ± 1 SD). The gravimetric water content was calculated as (wet soil mass—dry soil mass)/(dry soil mass); for the purpose of calculating gravimetric water content, we ignored the very small changes in organic matter content that occurred over the course of the experiment.

To determine organic matter content (g organic matter/g oven dry soil), subsamples (ca. 15 g) of oven-dry biocrusts were weighed and then ashed for 12 h in a muffle furnace (model 10-750-85, Fisher Scientific, Waltham, MA, USA) at 550°C. The amount of organic matter lost on ignition was calculated as the difference between the subsample weight and the ash weight, and organic matter content calculated relative to the subsample weight.

### Amplicon sequencing

We conducted amplicon sequencing to determine the composition of the bacterial and fungal communities. This analysis was done for two Set 1 samples (sample numbers 2 and 7) and two Set 2 samples (sample numbers 12 and 17) from each biocrust type. From each sample, a subsample of air-dried biocrust (ca. 150 g) was homogenized thoroughly by mixing in a clean Ziplok bag. We extracted genomic DNA in triplicate from 0.25 g of sample using the DNeasy Powersoil Pro kit (QIAGEN, Germantown, MD, USA). Samples were then combined. The V4 hypervariable region of the 16S rRNA gene was amplified in triplicates with 1X Phusion Green Hotstart High-Fidelity PCR Master Mix (Thermofisher Scientific, Fremont, CA, USA) using the primers 515F and 806R (Apprill et al., 2015; Parada et al., 2016) and the following PCR program: 95°C for 2 min, 15 cycles of 95°C for 30 s, 55°C for 30 s, and 72°C for 15 s, and used as a template in the subsequent tailing reaction with region-specific primers that included the Illumina flow cell adapter sequences and eight nucleotide barcodes. Amplicons were sequenced on an Illumina MiSeq instrument using a v2 300 cycle reagent kit (Illumina Inc., San Diego, CA, USA). Bacterial sequences were processed with QIIME 2-2022.2 (Bolyen et al., 2019). The paired-end reads were quality filtered, denoised, dereplicated, and chimera filtered using the DADA2 pipeline (Callahan et al., 2016). Taxonomy was assigned to the amplicon sequence variants (ASVs) using a trained Naïve Bayes classifier (SILVA 138; Quast et al., 2012) for the 16S rRNA V3–V4 hyper variable region using the q2-feature-classifier plugin. ASVs that accounted for less than 0.005% of the total sequences were removed. Additionally, taxa were excluded if they were not present in at least three samples, or were identified as chloroplast or mitochondrial sequences, leaving 1,010 ASVs. Feature tables were rarefied to 25,000 sequences for all subsequent community analyses.

ITS sequencing library preparation was similar to constructing 16S rRNA gene sequencing libraries. The primers 5.8S-Fun and ITS4-Fun (Taylor et al., 2016) were used in the two PCR steps under the following conditions: 95°C for 2 min; 20 cycles of 95°C for 30 s, 50°C for 30 s, and 72°C for 1 min. Amplicons were sequenced on an Illumina MiSeq instrument using a v2 500 cycle reagent kit (Illumina Inc., San Diego, CA, USA). Fungal sequences were processed with QIIME 2-2022.2 (Bolyen et al., 2019). Read one was quality filtered, denoised, dereplicated, and chimera filtered using the DADA2 pipeline. Taxonomy was assigned to the ASVs using the VSEARCH-based consensus taxonomy classifier plugin (Rognes et al., 2016) in QIIME2 and the UNITE database (Abarenkov et al., 2010). ASVs that were unassigned, accounted for less than 0.005% of the total sequences, or were present in less than three samples were removed, leaving 84 taxa. Feature tables were rarefied to 7,700 sequences for all subsequent community analyses.

A Bray–Curtis similarity matrix of the relative abundance data was used for permutational multivariate analysis of variance (PERMANOVA) and non-metric multi-dimensional scaling analysis (NMDS). Linear models were used to test for the effects and crust stage on richness and Shannon’s H. Data analysis was performed in Primer v7 and R (Clarke and Gorley, 2015; R Core Team, 2016).

## Results

### Community composition and cover

**FIGURE 1:**
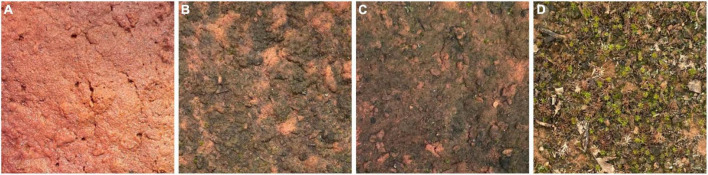
Examples of different greenhouse-grown biocrust soils, 13 weeks after inoculation. **(A)** Bare soil (control); **(B)** light cyanobacteria; **(C)** dark cyanobacteria; **(D)** moss-lichen. Each picture shows an area of approximately 5 cm × 5 cm.

The three biocrust treatments (light cyanobacteria, dark cyanobacteria, and moss-lichen) were characterized by distinctly different species mixtures and were visibly distinguishable from bare soil controls (Figure 1). Our light cyanobacteria biocrust was initially dominated by lightly pigmented filamentous cyanobacterial communities visually consistent with *Microcoleus*. Through time, dark pigmented, surface-bound cyanobacterial communities, mostly visually consistent with *Nostoc* became co-dominant. By the final time point, multiple mosses (particularly *Funaria hygrometrica*, and a *Bryum* sp.) frequently occurred but remained subdominant. The dark cyanobacteria biocrust followed a similar trajectory except that the dark-pigmented cyanobacterial communities became dominant more quickly and more completely. Mosses also colonized and remained subdominant, but an unidentified moss (likely Pottiaceae) was the most frequent and abundant. Our moss-lichen biocrust was dominated throughout by the moss *Syntrichia ruralis*. Light and dark cyanobacterial communities were the most frequent and abundant subdominants. Several lichens and additional mosses also commonly occurred in this treatment, including *Collema tenax*, *Cladonia pyxidata*, *F. hygrometrica*, and *Bryum argenteum*.

**FIGURE 2:**
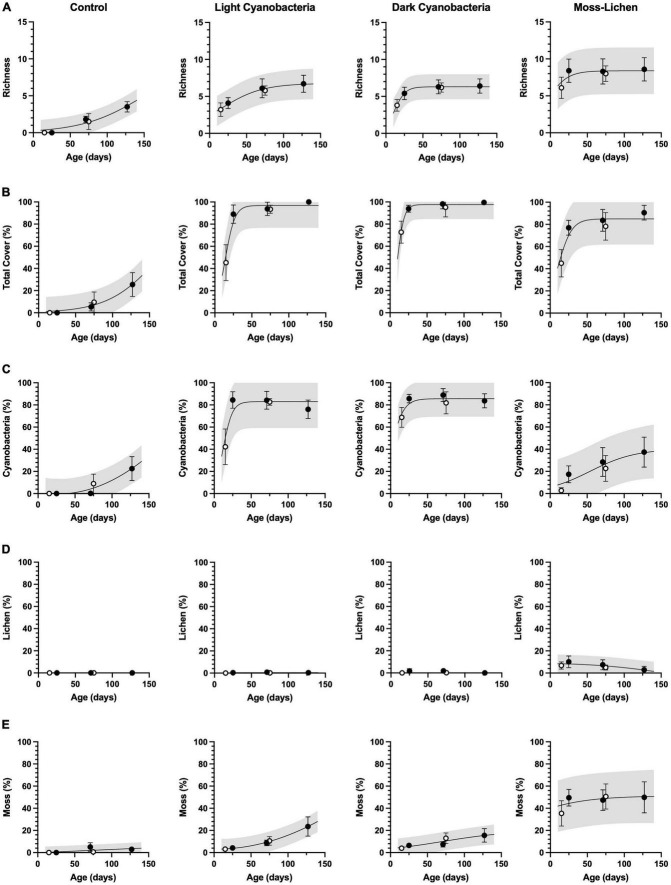
Cover type richness and community composition in relation to sample age (days since initial inoculation; *x* axis) and experimental treatment. **(A)** Richness is a count of the observed number of cover types. **(B)** Total cover is the sum of % cover by cyanobacteria **(C)**, lichen **(D)**, and moss **(E)**. Filled circles are inoculation “Set 1” (begun April 8), open circles are “Set 2” (begun June 3). Error bars are ± 1 SD on the observations. Lines are non-linear regressions fit to the data (either a sigmoid model, or a second-order polynomial if the sigmoid was a poor fit to the data or if the sigmoid parameters were not well-constrained by the data). Shading indicates 95% confidence intervals on the prediction band, i.e., where individual new measurements would likely fall, given the model and the data.

Community composition and total cover (Figure 2) changed with age as biocrusts developed following inoculation, and the developmental patterns were consistent between inoculation Set 1 (begun April 8) and Set 2 (begun June 3). Cover type richness (Figure 2A) in the control containers was still increasing even after 18 weeks (126 days), as inoculation by airborne spores occurred naturally and the moist substrate provided a favorable medium for growth. The light cyanobacteria biocrusts reached a plateau of about 5–7 cover types, similar to the dark cyanobacteria biocrusts, although the dark cyanobacteria achieved comparable cover type richness more rapidly. Moss-lichen biocrusts reached a plateau of 7–10 cover types within 10 weeks.

Total cover (Figure 2B) in the control containers (≈30%) was still increasing at the end of the experiment, mostly due to an increasing trend in cyanobacteria cover (Figure 2C) and a small amount of moss (Figure 2E) but essentially no lichen cover (Figure 2D). For both light cyanobacteria and dark cyanobacteria biocrusts, more than 90% total cover was achieved within 4 weeks, with cyanobacteria dominating (≈80% cover). Over the 18 weeks of observation, moss cover increased steadily in both light cyanobacteria and dark cyanobacteria biocrusts, reaching ≈20% by the end of the experiment. For the moss-lichen biocrusts, cover increased from ≈50% at 2 weeks, to ≈80% at 4 weeks, and ≈90% at 18 weeks. Community composition of the moss-lichen biocrusts changed substantially over the experiment, with cyanobacteria cover increasing from <10 to >30%, lichen cover decreasing from ≈10 to <5%, and moss cover increasing from ≈40 to ≈50%. There was also substantial variation among replicate containers in the community composition of the moss-lichen biocrusts; for example, at 18 weeks two containers had cyanobacteria cover <20% while two containers had cyanobacteria cover >50%; the two containers with the lowest cyanobacteria cover had the highest moss cover (>60%), while the two containers with the highest cyanobacteria cover had the lowest moss cover (<35%).

Sequencing data further elucidate the similarities and differences in composition of the “invisible” microbial communities across biocrust types (Supplementary Figure 2; note that full feature tables with taxonomic classification, by biocrust type and inoculation set, is available in the Figshare repository cited below, in section “Data availability statement”). These results complement our visual assessment of the macroscopic community elements (Figure 2). About 45% (395 of 907) of the bacterial taxa were common to all three biocrust types, but 5–10% of the bacterial taxa were unique to each biocrust type. Moss-lichen biocrusts had more unique bacterial taxa than either light or dark cyanobacteria biocrusts. The number of taxa shared between light and dark cyanobacteria biocrusts was higher than the number of taxa shared by either of those biocrust types and moss-lichen biocrusts. A majority (70% or 59 of 84) of fungi were common to all biocrust types. Moss-lichen biocrusts had more unique fungal taxa than either light or dark cyanobacteria biocrusts, and as with the bacterial taxa, the light and dark cyanobacterial biocrusts shared the most fungal taxa.

**FIGURE 3:**
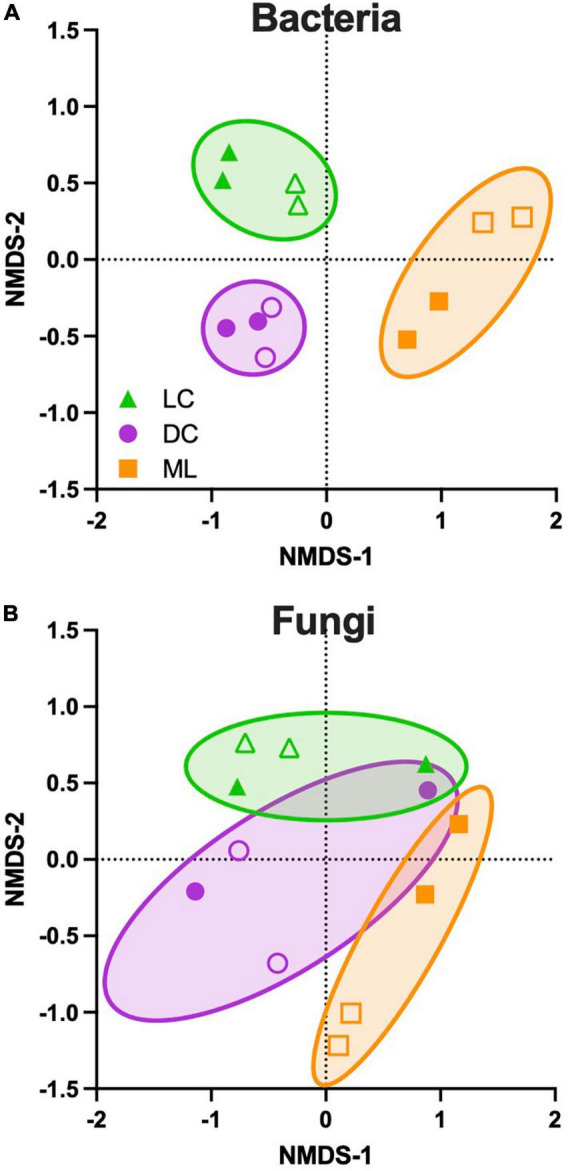
Ordination of microbial community composition across three biocrust types, based on non-metric multi-dimensional scaling (NMDS) conducted using relative abundance data. **(A)** Bacterial and **(B)** fungal taxa. Biocrust types represent a successional gradient: LC, early-successional light cyanobacteria biocrust; DC, mid-successional dark cyanobacteria biocrust; and ML, late-successional moss-lichen biocrusts. Filled symbols indicate samples from inoculation set 1 and open symbols indicate samples from inoculation set 2. Set 1 samples were about 8 weeks older (more developed) than set 2 samples. For bacteria, NMDS stress value = 0.07 and the permutational multivariate analysis of variance (PERMANOVA) ECV (estimated components of variation) is 0.52 for biocrust type, and 0.13 for inoculation set. For fungi, NMDS stress value = 0.14 and the ECV is 0.31 for biocrust type and 0.31 for inoculation set. Comparable results are obtained using ADONIS in the vegan package: for bacteria, 41% of the variance is explained by biocrust type, 10% by inoculation set, and 18% by biocrust type:inoculation set. For fungi, 31% of the variance is explained by biocrust type, 20% by inoculation set, and 9% by biocrust type:inoculation set.

Based on NMDS using relative abundance, the bacterial communities were distinct among biocrust types (PERMANOVA *F* = 4.9, *p* = 0.002). There was a clear separation of light and dark cyanobacteria biocrusts from moss-lichen biocrusts along the first ordination axis, and similarly a clear separation of light and dark cyanobacteria biocrusts along the second ordination axis (Figure 3A). For fungal taxa (Figure 3B), the separation among biocrust types was not quite as distinct, although the differences among biocrust types were still statistically significant (PERMANOVA *F* = 2.1, *p* = 0.017). For both bacterial and fungal taxa, differences between Set 1 and Set 2 samples were not significant (all *p* > 0.05) when the analysis was conducted using both biocrust type and inoculation set as factors, suggesting that by the end of the experiment both inoculation sets had developed along similar trajectories of community composition.

Despite differences in microbial community composition, microbial diversity was similar across different successional stages. For both bacterial and fungal taxa, neither richness (*F* = 1.1, *p* = 0.36 and *F* = 0.87, *p* = 0.45, respectively) nor Shannon’s H (*F* = 3.4, *p* = 0.08 and *F* = 2.5, *p* = 0.14, respectively) were significantly different among biocrust types. These results are in contrast to the community composition surveys, which documented higher cover type richness in the moss-lichen biocrusts than either the light or dark cyanobacteria biocrusts.

Together, the sequencing results show how microbial community composition, but not microbial diversity, varied among biocrust types representing a successional gradient, from early-successional light cyanobacteria biocrusts to mid-successional dark cyanobacteria biocrusts and late-successional moss-lichen biocrusts. While almost half of the microbial taxa were common to biocrust types across all successional stages, there were unique taxa associated with each biocrust type.

### Weekly flux measurements

Beginning in late May, and throughout the remainder of the experiment, we measured CO_2_ and CH_4_ fluxes from all experimental samples on a weekly basis.

#### Blanks

Across all measurement dates, mean CO_2_ efflux from blank containers was slightly positive (μ ± 1 SD = 0.005 ± 0.006 μmol CO_2_ m^–2^ s^–1^, *n* = 45) but low (<1%) compared to the biocrust CO_2_ fluxes, and smaller than our estimated minimum detectable CO_2_ flux. However, the 95% confidence interval on the mean (0.003–0.006 μmol CO_2_ m^–2^ s^–1^) did not actually include zero. The CH_4_ efflux from blank containers was also positive (μ ± 1 SD = 0.006 ± 0.004 nmol CH_4_ m^–2^ s^–1^, *n* = 46), and similar in magnitude to the CH_4_ flux from both biocrusts and control samples, but larger than our estimated minimum detectable CH_4_ flux. As with CO_2_, the 95% confidence interval on the mean blank CH_4_ flux (0.005–0.007 nmol CH_4_ m^–2^ s^–1^) did not include zero. Thus, for both CO_2_ and CH_4_, we measured a small but statistically significant non-zero background flux from the empty containers used as blanks.

#### CO_2_

**FIGURE 4:**
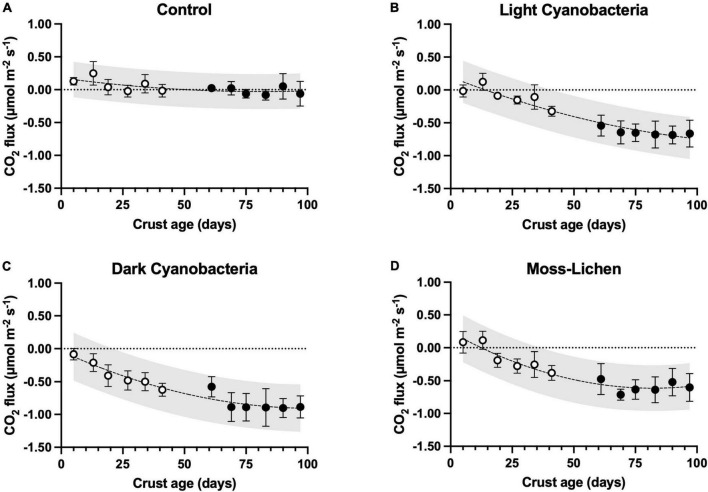
Carbon dioxide fluxes in relation to sample age (days since initial inoculation; *x* axis), by experimental treatment. **(A)** Control samples (containers with non-inoculated substrate), **(B)** light cyanobacteria biocrusts, **(C)** dark cyanobacteria biocrusts, and **(D)** moss-lichen biocrusts. Best-fit curves are second-order polynomials, and shading indicates 95% prediction band. Filled symbols are samples from inoculation “Set 1” (begun April 8), while open symbols are from “Set 2” (begun June 3); error bars are ± 1 SD on the observations.

CO_2_ fluxes varied among experimental treatments, and in relation to age, or the number of days since inoculation (Figure 4). We measured small rates of CO_2_ efflux (i.e., cellular respiration > photosynthetic uptake) from young, bare-soil control samples, which had not been inoculated. But in parallel with the increase in cover type richness and total cover that occurred over time in the control samples, after 8 or 10 weeks many control samples were observed to be taking up small amounts of CO_2_ as photosynthetic organisms became established. For each of the inoculated biocrusts, CO_2_ fluxes hovered around zero (slightly positive or negative, depending on the balance between respiration and photosynthesis) in the first few weeks after inoculation. Then, as biocrusts developed (Figure 2), they took up progressively more CO_2_ and from 5 weeks onward, the mean flux measured for each biocrust treatment consistently indicated net CO_2_ uptake. At 14 weeks, the highest rate of net uptake was by the dark cyanobacteria biocrusts (μ ± 1 SD = −0.885 ± 0.169 μmol CO_2_ m^–2^ s^–1^, *n* = 10), with slightly lower rates of net uptake by the light cyanobacteria (μ ± 1 SD = −0.663 ± 0.205 μmol CO_2_ m^–2^ s^–1^, *n* = 10) and moss-lichen (μ ± 1 SD = −0.601 ± 0.211 μmol CO_2_ m^–2^ s^–1^, *n* = 10) biocrusts.

Mixed model analysis (see Supplementary Table 1 for the ANOVA table) of the weekly CO_2_ flux measurements revealed significant treatment (biocrust type) effects that interacted with biocrust age and air temperature (Supplementary Figure 3), along with effects of other environmental factors. Differences among treatments increased in older biocrusts and at warmer temperatures: at an age of 25 days and temperature of 18°C, all pairwise differences between biocrusts and controls were significant at 95% confidence, but biocrusts were not significantly different from each other. At an age of 25 days and a temperature of 30°C, all pairwise differences were statistically significant with the exception of light cyanobacteria vs. moss-lichen biocrusts. At an age of 100 days and a temperature of 30°C, all pairwise differences between treatments were statistically significant.

#### CH_4_

**FIGURE 5:**
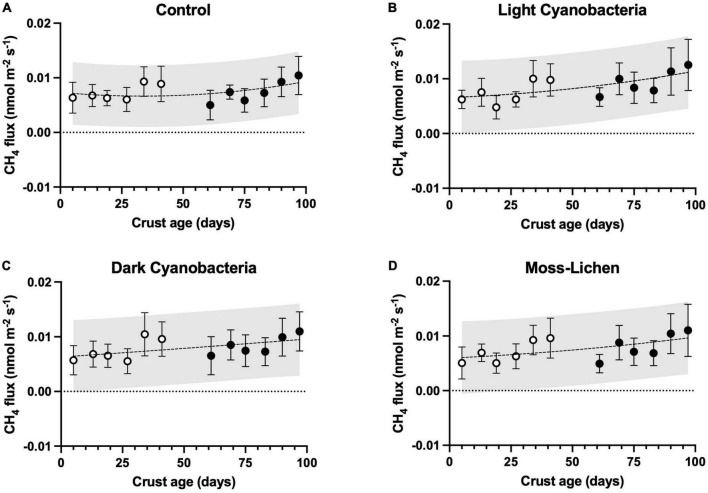
Methane fluxes in relation to sample age (days since initial inoculation; *x* axis), by experimental treatment. **(A)** Control samples (containers with non-inoculated substrate), **(B)** light cyanobacteria biocrusts, **(C)** dark cyanobacteria biocrusts, and **(D)** moss-lichen biocrusts. Best-fit curves are second-order polynomials, and shading indicates 95% prediction band. Filled symbols are samples from inoculation “Set 1” (begun April 8), while open symbols are from “Set 2” (begun June 3); error bars are ± 1 SD on the observations.

We consistently measured CH_4_ efflux from our samples, including controls, suggesting very small rates of CH_4_ production (μ ± 1 SD = 0.008 ± 0.004 nmol m^–2^ s^–1^, across all sampling dates and treatments; Figure 5). Based on our ANCOVA model analysis (Supplementary Table 2), there were significant treatment effects (*p* < 0.001). Specifically, both light cyanobacteria biocrusts (difference ± 1 SE = 0.0012 ± 0.0003 nmol m^–2^ s^–1^, *p* < 0.001) and dark cyanobacteria biocrusts (difference ± 1 SE = 0.0007 ± 0.0003 nmol m^–2^ s^–1^, *p* = 0.04) had modestly higher (10–20%) rates of CH_4_ efflux than controls (μ ± 1 SD = 0.0074 ± 0.0029 nmol m^–2^ s^–1^). However, CH_4_ efflux from moss-lichen biocrusts did not differ from controls (difference ± 1 SE = 0.0003 ± 0.0003 nmol m^–2^ s^–1^, *p* = 0.54).

From the ANCOVA analysis of the CH_4_ flux data, we identified a number of statistically significant covariates (Supplementary Table 2). For example, there was a very small but statistically significant trend of increasing CH_4_ efflux as biocrusts aged (slope ± 1 SE = 0.0195 ± 0.0033 pmol m^–2^ s^–1^ d^–1^, *p* < 0.001; note prefix of pico rather than nano), and the quadratic air temperature effect explained a 0.001 nmol m^–2^ s^–1^ difference in CH_4_ flux between 20 and 25°C. However, other environmental covariates (PPFD and soil moisture) individually explained relatively little of the variation in CH_4_ flux across samples or sampling dates, although the model as a whole explained roughly two-thirds of the overall variance.

### End-of-experiment flux measurements

At the conclusion of the experiment, we conducted a final set of flux measurements on all samples, including blanks and controls as well as the three biocrust community types. At this time, the inoculation Set 1 samples were 19 weeks old and the Set 2 samples were 11 weeks old. In contrast to the weekly measurements described above, these measurements were made by a different individual and using a slightly different measurement system enabling simultaneous measurement of CO_2_, CH_4_, and N_2_O fluxes. For these reasons, these data are analyzed separately from the weekly flux measurements.

#### CO_2_

**FIGURE 6:**
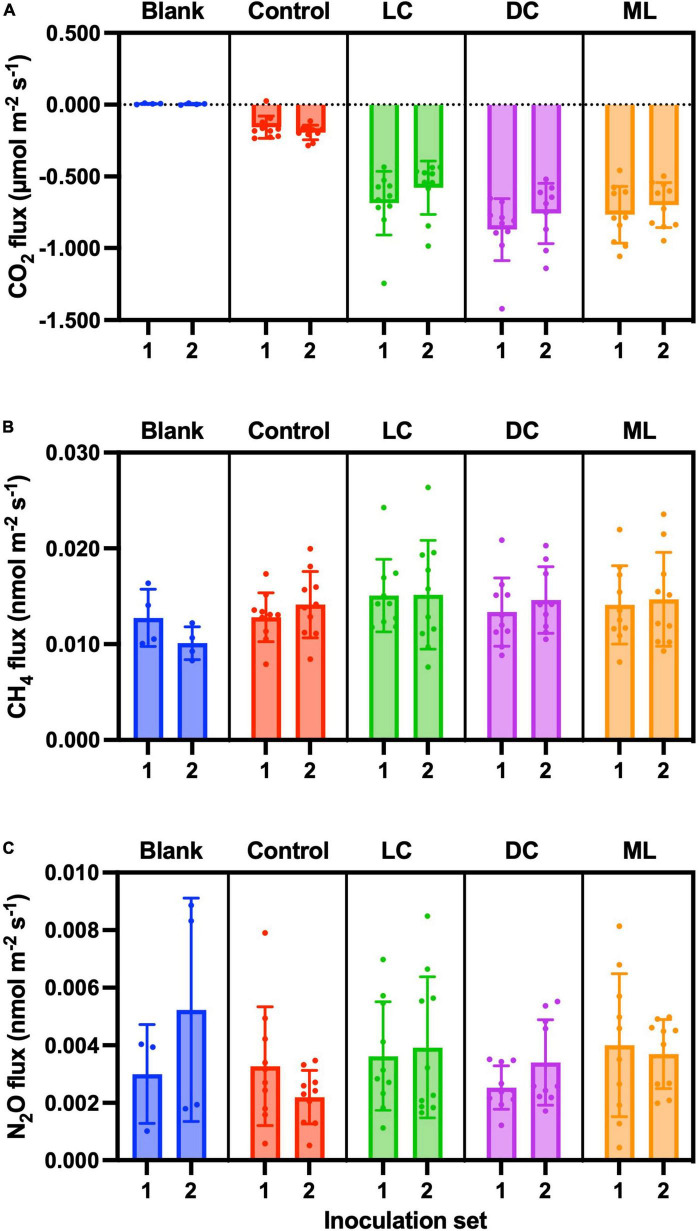
End-of-experiment fluxes of **(A)** CO_2_, **(B)** CH_4_, and **(C)** N_2_O. Blank measurements consisted of an empty plastic container within the measurement cuvette and Controls were containers with non-inoculated substrate. Biocrust types represent a successional gradient: LC, early-successional light cyanobacteria biocrust; DC, mid-successional dark cyanobacteria biocrust; and ML, late-successional moss-lichen biocrusts. Set 1 microcosms were inoculated on April 8 and therefore older (more developed) than the Set 2 microcosms that were inoculated on June 3.

CO_2_ fluxes were essentially zero from the blank containers (μ ± 1 SD = 0.005 ± 0.005 μmol CO_2_ m^–2^ s^–1^, *n* = 8), but we measured a small amount of CO_2_ uptake by the control samples (μ ± 1 SD = −0.175 ± 0.066 μmol CO_2_ m^–2^ s^–1^, *n* = 20). The dark cyanobacteria biocrusts had the highest rates of uptake (μ ± 1 SD = −0.818 ± 0.216 μmol CO_2_ m^–2^ s^–1^, *n* = 19), followed by moss-lichen (μ ± 1 SD = −0.734 ± 0.178 μmol CO_2_ m^–2^ s^–1^, *n* = 20) and then light cyanobacteria (μ ± 1 SD = −0.633 ± 0.208 μmol CO_2_ m^–2^ s^–1^, *n* = 20) biocrusts (Figure 6A).

The final ANCOVA model for these data (Supplementary Table 3) included only treatment effects (biocrust type), air temperature (not significant), and an interaction of treatment and air temperature. This interaction was driven by the fact that pairwise differences between biocrusts and controls, and also between biocrust types, were amplified at higher temperatures (Supplementary Figure 4). Pairwise differences between biocrust types were generally significant at *p* < 0.05, except that at cooler temperatures (air temperature ≤ 21°C), differences in CO_2_ flux between moss-lichen and dark cyanobacteria biocrusts were not significant, and at warmer temperatures (air temperature ≥ 24°C) differences between moss-lichen and light cyanobacteria biocrusts were not significant. Surprisingly, our mixed model analysis did not indicate a significant difference between Set 1 and Set 2 biocrusts, and this effect was therefore dropped from the model. The environmental covariates PPFD and soil moisture were also not significant in this analysis and were dropped.

Overall, these results are largely consistent with the weekly CO_2_ flux measurements, which indicated that (1) all biocrusts were actively taking up CO_2_ at rates that were elevated above the control samples; (2) dark cyanobacteria biocrusts had somewhat higher rates of CO_2_ uptake than light cyanobacteria or moss-lichen biocrusts; and (3) a strong interaction effect between treatment and air temperature resulted in more pronounced treatment differences in CO_2_ flux at warmer temperatures.

The CO_2_ fluxes measured under sunlit conditions indicate that net photosynthesis by the dark cyanobacteria biocrusts was about 10% higher than by the moss-lichen biocrusts, and 25% higher than by the light cyanobacteria biocrusts. By comparison, the CO_2_ fluxes measured in the dark show that the moss-lichen biocrusts had the highest rates (μ ± 1 SD = 0.164 ± 0.099 μmol CO_2_ m^–2^ s^–1^, *n* = 20) of ecosystem respiration—more than double the rates for either the dark cyanobacteria (μ ± 1 SD = 0.079 ± 0.064 μmol CO_2_ m^–2^ s^–1^, *n* = 20) or the light cyanobacteria (μ ± 1 SD = 0.058 ± 0.043 μmol CO_2_ m^–2^ s^–1^, *n* = 20) biocrusts (Table 1). As a result of these differences, gross ecosystem photosynthesis was about 30% higher for both the moss-lichen and dark cyanobacteria biocrusts than the light cyanobacteria biocrusts (Table 1).

**TABLE 1:** Measured rates (μ ± 1 SD) of net photosynthesis (here, a positive flux indicates photosynthetic uptake), ecosystem respiration, and gross ecosystem photosynthesis in relation to biocrust type and inoculation set.

Biocrust type	Set	Net photosynthesis	Respiration	Gross photosynthesis
		(μmol m^–2^ s^–1^)	(μmol m^–2^ s^–1^)	(μmol m^–2^ s^–1^)
Light cyanobacteria	1	0.687 ± 0.223	0.066 ± 0.045	0.753 ± 0.241
	2	0.579 ± 0.187	0.051 ± 0.042	0.630 ± 0.215
Dark cyanobacteria	1	0.871 ± 0.217	0.075 ± 0.070	0.946 ± 0.239
	2	0.758 ± 0.211	0.083 ± 0.061	0.839 ± 0.244
Moss-lichen	1	0.768 ± 0.199	0.197 ± 0.108	0.964 ± 0.217
	2	0.700 ± 0.158	0.131 ± 0.082	0.831 ± 0.200

Ecosystem respiration was measured on darkened samples, with the chamber system covered by a light-blocking box. Gross ecosystem photosynthesis was calculated as ecosystem respiration—net photosynthesis. Measurements were conducted at the end of the experiment, at which time “Set 1” samples were 19 weeks old and “Set 2” samples were 11 weeks old (n = 10 samples of each biocrust type per set).

#### CH_4_

We measured CH_4_ efflux (Figure 6B) from the blank containers that was not only non-zero (μ ± 1 SD = 0.011 ± 0.003 nmol CH_4_ m^–2^ s^–1^, *n* = 8) but also somewhat larger than previous blank measurements (see “Blanks,” above). CH_4_ efflux from the control samples (μ ± 1 SD = 0.013 ± 0.003 nmol CH_4_ m^–2^ s^–1^, *n* = 20) was similar in magnitude to blanks. CH_4_ efflux from light cyanobacteria biocrusts (μ ± 1 SD = 0.015 ± 0.005 nmol CH_4_ m^–2^ s^–1^, *n* = 20) was marginally higher than from dark cyanobacteria (μ ± 1 SD = 0.014 ± 0.004 nmol CH_4_ m^–2^ s^–1^, *n* = 19) or moss-lichen (μ ± 1 SD = 0.014 ± 0.004 nmol CH_4_ m^–2^ s^–1^, *n* = 20) biocrusts. The treatment effect was not significant in our ANCOVA model (Supplementary Table 4). The lack of a significant biocrust treatment effect in these data, compared with the weekly measurements (see “CH_4_”), may be the result of the smaller number of end-of-experiment measurements and hence lower statistical power. In fact, the only factor that was significant in our model was air temperature, as measured CH_4_ flux covaried positively with temperature, approximately doubling from 0.010 nmol CH_4_ m^–2^ s^–1^ at 18°C to 0.024 nmol CH_4_ m^–2^ s^–1^ at 30°C.

These results are qualitatively similar to those obtained from the weekly measurement, in that (1) in both cases, blank container CH_4_ fluxes were non-zero, and control sample CH_4_ fluxes were similar in magnitude to blanks and (2) differences between biocrust treatments and controls were negligible.

#### N_2_O

As with CH_4_, measured N_2_O fluxes were extremely small but consistently positive (Figure 6C). For blank containers, the mean N_2_O efflux (μ ± 1 SD = 0.004 ± 0.003 nmol N_2_O m^–2^ s^–1^, *n* = 7; 95% confidence interval on the mean: 0.001–0.007 nmol N_2_O m^–2^ s^–1^) was non-zero. N_2_O efflux from the control samples (μ ± 1 SD = 0.003 ± 0.002 nmol N_2_O m^–2^ s^–1^, *n* = 20) was similar to that from the blanks. Notably these fluxes are comparable in magnitude to our estimated minimum detectable flux. Mean N_2_O efflux from all biocrust types was slightly higher than from control samples. In our model-based analysis (Supplementary Table 5), the biocrust type treatment was only marginally significant (*p* = 0.08), and the only significant pairwise difference between treatments was for moss-lichen biocrusts (μ ± 1 SD = 0.004 ± 0.002 nmol N_2_O m^–2^ s^–1^, *n* = 20) vs. control samples (difference ± 1 SE = 0.0011 ± 0.0005 nmol N_2_O m^–2^ s^–1^). While there was a strong and significant positive relationship between measured N_2_O flux and PPFD, other factors were not significant.

### Soil organic matter content

**FIGURE 7:**
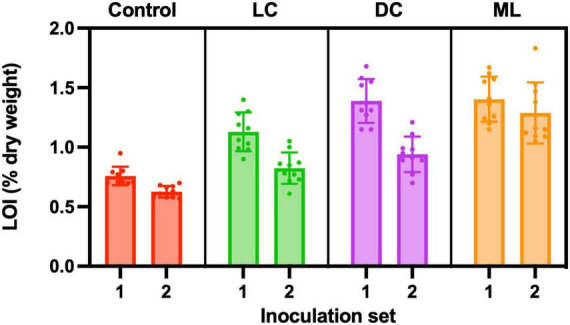
Organic matter content (% dry weight) measured using loss-on-ignition (LOI). Control containers were filled with the same soil used in light cyanobacteria (LC), dark cyanobacteria (DC) and moss-lichen (ML) biocrust containers, but were not inoculated. Set 1 microcosms were inoculated on April 8 and therefore older (more developed) than the Set 2 microcosms that were inoculated on June 3.

Control samples had an organic matter content of about 0.7%, and this was lower than any of the experimental biocrusts. Moss-lichen biocrusts had more organic matter than dark cyanobacteria biocrusts, which in turn had more organic matter than light cyanobacteria biocrusts, which in turn had more organic matter than controls (Figure 7). Based on a two-way ANOVA, there were significant differences in organic matter content among experimental treatments (*p* < 0.001) and between inoculation sets (*p* < 0.001). There was also a significant interaction (*p* < 0.01) between treatment and inoculation set, as the difference between the older (higher organic matter content) Set 1 and the younger (lower organic matter content) Set 2 was larger for light cyanobacteria and dark cyanobacteria samples, and smaller for moss-lichen and control samples. Accounting for differences in inoculum carbon added to each biocrust type, these data suggest that the highest rates of C accumulation occurred in the dark cyanobacteria biocrusts. At the level of individual samples, there was a modest correlation between organic matter content and the CO_2_ flux measured at the end of the experiment for both the Set 1 (*r* = −0.71) and Set 2 (*r* = −0.73) samples, but this was driven by differences across biocrust types, and the correlation was poor within each type (mean *r* ± 1 SD = −0.19 ± 0.27).

Based on the median differences in organic matter content between Sets 1 and 2, the age difference of 56 days between Sets 1 and 2, and assuming that organic matter is 50% C, we estimate that the dark cyanobacteria biocrusts were accumulating C at a net rate of approximately 0.5 g C m^–2^ day^–1^, vs. 0.4 g C m^–2^ day^–1^ for light cyanobacteria biocrusts, 0.3 g C m^–2^ day^–1^ for moss-lichen biocrusts, and 0.1 g C m^–2^ day^–1^ for control samples. These rates of C accumulation are essentially consistent with the CO_2_ flux measurements, as 0.5 g C m^–2^ day^–1^ equates to about 1 μmol m^–2^ s^–1^ over a 12 h photoperiod. These rates are also much higher than would be expected under field conditions, but were made possible by the regular watering regime, which allowed many years of biocrust development to occur within a matter of months. The fact that C accumulation, relative to measured end-of-experiment net CO_2_ uptake rate, was higher in the dark cyanobacteria and light cyanobacteria biocrusts, and lower in the moss-lichen biocrusts, is consistent with the higher measured rates of ecosystem respiration from the moss-lichen biocrusts (Table 1). This may be due to the increased structural complexity—and associated biomass—of the dominant bryophyte community (Figure 2).

## Discussion

Our objective was to better understand relationships between biocrust function and successional stage, and how these relationships varied over space and time. We measured trace gas fluxes (CO_2_, CH_4_, and N_2_O) along a successional gradient of biocrust community types, and investigated whether these fluxes varied as each community matured over the course of an 18-week experiment. By conducting this experiment using well-watered biocrusts grown in a greenhouse setting, we were able to manipulate the successional stage of each sample and accelerate their development, or recovery of form and function from a disturbed state, so that by the end of the experiment we had mature examples of each successional stage.

### CO_2_ fluxes and C accumulation

We had predicted that net CO_2_ uptake would increase as a function of successional stage and development over the course of the experiment. These predictions were based on inherent differences related to the dominant communities associated with each successional stage, and expected increases in photosynthetic biomass of individual crusts over time. Indeed, our community composition surveys documented how the relative abundance of different taxa varied among biocrust types (Figure 2), and how these communities matured over the course of the experiment. At the same time as the biocrust communities developed, we saw concurrent increases in net photosynthetic uptake, with all biocrust communities becoming rapidly established as net C sinks within a few weeks of inoculation (Figure 4). By the end of our experiment, rates of net CO_2_ uptake by mid-successional dark cyanobacteria biocrusts were about 25% higher than early-successional light cyanobacteria biocrusts and 10% higher than late-successional moss-lichen biocrusts (Figure 6). Rates of C accumulation (see “Soil organic matter content,”) were also highest in the mid-successional dark cyanobacteria biocrusts and lowest in the late-successional moss-lichen biocrusts, probably because of the much higher respiratory losses of the moss-lichen biocrusts (Table 1) compared to either the light or dark cyanobacteria biocrusts. This could be explained by the greater structural biomass associated with moss-lichen biocrusts and the need to invest in both the production and maintenance of these structural tissues. The carbon economies of different successional stages are very different: cyanobacteria invest C in polysaccharides and the growth of new photosynthetic cells. By comparison, mosses and lichens both invest some of the C fixed through photosynthesis in structural tissues—stems in the case of mosses, and thalli in the case of lichens—which also require C investments in maintenance.

These results do not support our prediction that the late-successional moss-lichen biocrusts would have the highest rates of CO_2_ uptake. Intriguingly, however, the successional patterns we observed for biocrust CO_2_ exchange and C accumulation are highly analogous to the classical model of forest succession, in which middle-age forests are considered to be more productive than younger or older forests (Tang et al., 2014), and declines in net primary productivity in old forests that have been attributed to the ever-increasing burden of respiration (Kira and Shidei, 1967; Odum, 1969; but see Ryan et al., 1997). Overall, our results show how biocrust function varies as a continuous outcome of development and succession.

Several previous studies have measured net photosynthesis of different biocrust community types and characterized the sensitivity of photosynthesis to environmental factors. For example, Tamm et al. (2018) found that chlorolichen biocrusts had maximum rates of net photosynthesis that were 40% higher either cyanobacteria biocrusts or moss biocrusts. The photosynthetic temperature optimum was about 15°C warmer for cyanobacteria biocrusts than for either of the other two biocrust types. In contrast, Feng et al. (2014) found that moss biocrusts had maximum rates of net photosynthesis that were more double those of cyanobacterial or lichen biocrusts. However, cyanobacteria biocrusts again had the warmest temperature optima (see also Grote et al., 2010). We found a strong interaction effect of treatment and air temperature on the measured CO_2_ flux (Supplementary Figures 3, 4), because there was substantial enhancement of CO_2_ uptake at warmer temperatures for dark cyanobacteria biocrusts but little or no enhancement of CO_2_ uptake at warmer temperatures for moss-lichen biocrusts. Our data therefore add to understanding of how abiotic factors interact with biocrust successional stage to influence biocrust function.

### CH_4_ fluxes

Fluxes of CH_4_ are only sparingly documented in biocrusts and are understudied in general in dryland soils. Most studies point to CH_4_ consumption (methanotrophy) by dryland soils, but there is some evidence of the potential for CH_4_ production (methanogenesis) to occur under the right conditions. Lafuente et al. (2020) measured CH_4_ consumption of up to 1.45 nmol m^–2^ s^–1^ by biocrust-covered soils in a semiarid Mediterranean dryland. Durán et al. (2021) measured CH_4_ uptake by a range of moss- and lichen-dominated cryptogamic soils in Antarctica, although rates of CH_4_ uptake were actually 2–3 × higher in bare soil with no cryptogams. Working in Israel’s Negev Desert, Angel and Conrad (2009) measured CH_4_ uptake (consumption) at rates of 0.45–0.92 nmol m^–2^ s^–1^ by undisturbed soils in an arid environment, but found no evidence of uptake by disturbed soils in the arid environment, or by soils in a hyperarid environment. Notably, their data also show that in biocrust soils, methane oxidation occurs only below the soil surface, rather within the biocrust layer itself. More generally, desert soils have been consistently shown to be locations of CH_4_ consumption (Striegl et al., 1992; Hou et al., 2012). However, Aschenbach et al. (2013) and Angel et al. (2012) found that when incubated as a slurry in anoxic conditions, biocrust soils from many different dryland environments were capable of producing CH_4_.

Based on these previous studies, we had predicted that biocrust CH_4_ fluxes would be more strongly controlled by environmental factors—soil moisture and temperature—than by biological factors such as community composition, e.g., as driven by biocrust development or successional stage. Indeed, we expected that all crust types were equally capable of CH_4_ production or consumption, but that depending on how long it had been since irrigation occurred, drier biocrust samples would probably consume CH_4_ while wetter, recently-irrigated samples might produce CH_4_. In fact, however, we consistently measured very low rates of CH_4_ efflux (≈ 0.01 nmol m^–2^ s^–1^), and never CH_4_ consumption, by each of our biocrust communities, including controls, and regardless of soil moisture status. We note that we also observed measurable net efflux of CH_4_ from the empty containers that were measured each week as blank samples; we did not find strong evidence of CH_4_ fluxes from any biocrust type that were substantially higher than what we measured from blanks. This suggests that metabolic processes in the soil that involve CH_4_ were most likely occurring at rates near the detection limit of our measurement system, or were masked by other artifacts (although previous studies documented rates of consumption that should have been detectable with our system). One reason we may have observed low CH_4_ production is because our entire experiment was conducted under oxic conditions. This was appropriate for our goals because most biocrust habitats are prevailingly oxic, most of the time. Any CH_4_ production that did occur was likely confined to anoxic microsites within the biocrusts, conducive to anaerobes, but we acknowledge that the limited availability of anoxic microsites is a possible explanation for the low rates of CH_4_ production we observed.

### N_2_O fluxes

Biocrusts and other cryptogamic mats are known to fix atmospheric N, some of which may then be lost back to the atmosphere in a variety of gaseous forms (NH_3_, N_2_O, NO, HONO, and N_2_) through processes of nitrification and denitrification; they thus play a key role in the global N cycle and are potentially important sources of the greenhouse gas N_2_O (Lenhart et al., 2015; Weber et al., 2015; Barger et al., 2016). We predicted that we would be able to measure detectable N_2_O efflux that would be positively associated with successional stages that have a greater prevalence of N-fixing organisms and hence greater N availability. While we measured N_2_O efflux from each of the three biocrust treatments, the fluxes were extremely small and, with the exception of moss-lichen biocrusts, not statistically different from blanks or control treatments: fluxes averaged around 0.003 nmol m^–2^ s^–1^ for all treatments. Lafuente et al. (2020) measured somewhat larger biocrust N_2_O fluxes (mean values up to 0.005 nmol m^–2^ s^–1^) in a field experiment conducted in a Mediterranean dryland, but noted high temporal variability across measurements. They also observed that climatic manipulations (warming and rainfall exclusion) tended to reduce N_2_O fluxes (relative to controls) in some seasons and increase N_2_O fluxes in other seasons. By comparison, substantially larger N_2_O fluxes were measured from desert biocrust soils in China (0.09 nmol m^–2^ s^–1^; Jia et al., 2018) and Utah (0.02 nmol m^–2^ s^–1^ for early successional communities, and 0.17 nmol m^–2^ s^–1^ for late successional communities; Barger et al., 2013), while 500× larger fluxes (maximum of 1.4 nmol m^–2^ s^–1^; Brankatschk et al., 2013) can be inferred from denitrification assays conducted on biocrust soil samples from dunes in central Europe. Notably, the study by Brankatschk et al. (2013) found that there was a strong relationship between N cycling and the stage of biocrust soil development, with high abundance of functional genes for, and potential enzyme activity of, N fixation, nitrification, and denitrification characteristic only of well-developed biocrusts. This can be attributed to the increased abundance of N-fixing cyanobacteria and lichens in later-successional biocrusts (Barger et al., 2016; Rosentreter et al., 2016).

Our results—showing N_2_O fluxes that were non-zero but that can hardly be distinguished from control treatments—are therefore surprising, given that Antoninka et al. (2016) showed substantial N-fixation rates in similar greenhouse-grown biocrusts, suggesting there is no overall N shortage. However, we know little about the development of ammonia-oxidizer communities in biocrusts, which may regulate the supply of nitrate for denitrifiers. Our samples were possibly limited in nitrate specifically, leading to little substrate for denitrification. This explanation is plausible because nitrate is prone to leaching, which could have occurred as a result of our twice-daily watering regime. The limited availability of anoxic microsites that would have favored denitrification is also a possible explanation for the low rates of N_2_O production we observed. Alternatively, our low rates may have been due to the relative shallowness of our samples. Most other studies used cores of 5–20 cm depth or, field-installed collars with no defined depth. Therefore, most studies measured fluxes from a greater volume of soil underlying the biocrusts than we did, whereas we were more focused specifically on fluxes from the biocrust itself.

## Conclusion

In a greenhouse experiment, we studied the changes in community composition and structure as different biocrust types developed, from inoculation to 18 weeks of age. The biocrust types represented a successional gradient, from bare soil, to early-successional light cyanobacteria, mid-successional dark cyanobacteria, and late-successional moss-lichen communities. For all biocrust types, cover type richness increased as biocrusts developed over the course of the experiment, and cover type richness tended to be highest in the moss-lichen biocrusts. Bacterial and fungal community composition varied significantly among different biocrust types, but microbial richness did not. We conducted regular measurements of trace gas fluxes (CO_2_, CH_4_, and N_2_O) and found that within a few weeks of inoculation, photosynthesis substantially exceeded cellular respiration and measurable net CO_2_ uptake was occurring in all biocrust community types, but not in bare soil controls. Continued biocrust development was associated with further increases in photosynthetic uptake of CO_2_ by each biocrust type. Our predictions were not completely supported for CO_2_, as the mid-successional dark cyanobacteria biocrusts had higher rates of CO_2_ uptake than the late-successional moss-lichen biocrusts, as well as the early-successional light cyanobacteria biocrusts. Although our biocrust samples were sources of both CH_4_ and N_2_O, the measured fluxes were small compared to what has been reported in previous studies; measured CH_4_ efflux from biocrusts was only marginally higher than from control samplesx and blanks, while measured N_2_O efflux could hardly be distinguished from controls or blanks. Our predictions were therefore not supported for either CH_4_ or N_2_O. Our approach of experimentally growing replicated biocrust communities under controlled, near-optimal greenhouse conditions provides unique opportunities to improve understanding of the biological and environmental controls on biocrust greenhouse gas fluxes. Our results show how biocrust function varies in relation to both development and successional stage. In a follow-on paper we will present spectral measurements from the same experiment, and link the developmental and successional changes in reflectance to the CO_2_ fluxes presented here.

## Data availability statement

The datasets presented in this study can be found in online repositories. The names of the repository/repositories and accession number(s) can be found below: https://doi.org/10.6084/m9.figshare.20060927.v2.

## Author contributions

AR, JL, MB, MC, GWK, MS, and DT designed research. AR, JL, DB, MC, and GWK designed flux measurement system. GVK, KT, JL, MB, and AS conducted measurements. MH conducted sequencing. AR, JL, MB, JB, and MH analyzed the data. AR drafted the manuscript. All authors provided feedback on the manuscript.
