# Supplementary material for: Soil-atmosphere fluxes of CO2, CH4, and N2O across an experimentally-grown, successional gradient of biocrust community types
Source: Front Microbiol. 2022 Sep 26;13:979825. doi: 10.3389/fmicb.2022.979825 (PMC9549369; doi:10.3389/fmicb.2022.979825)
Supplement: Supplementary file 1 [file Data_Sheet_1.pdf]

frontiers

Supplementary Material

1 Supplementary Figures and Tables

1.1 Supplementary Figures

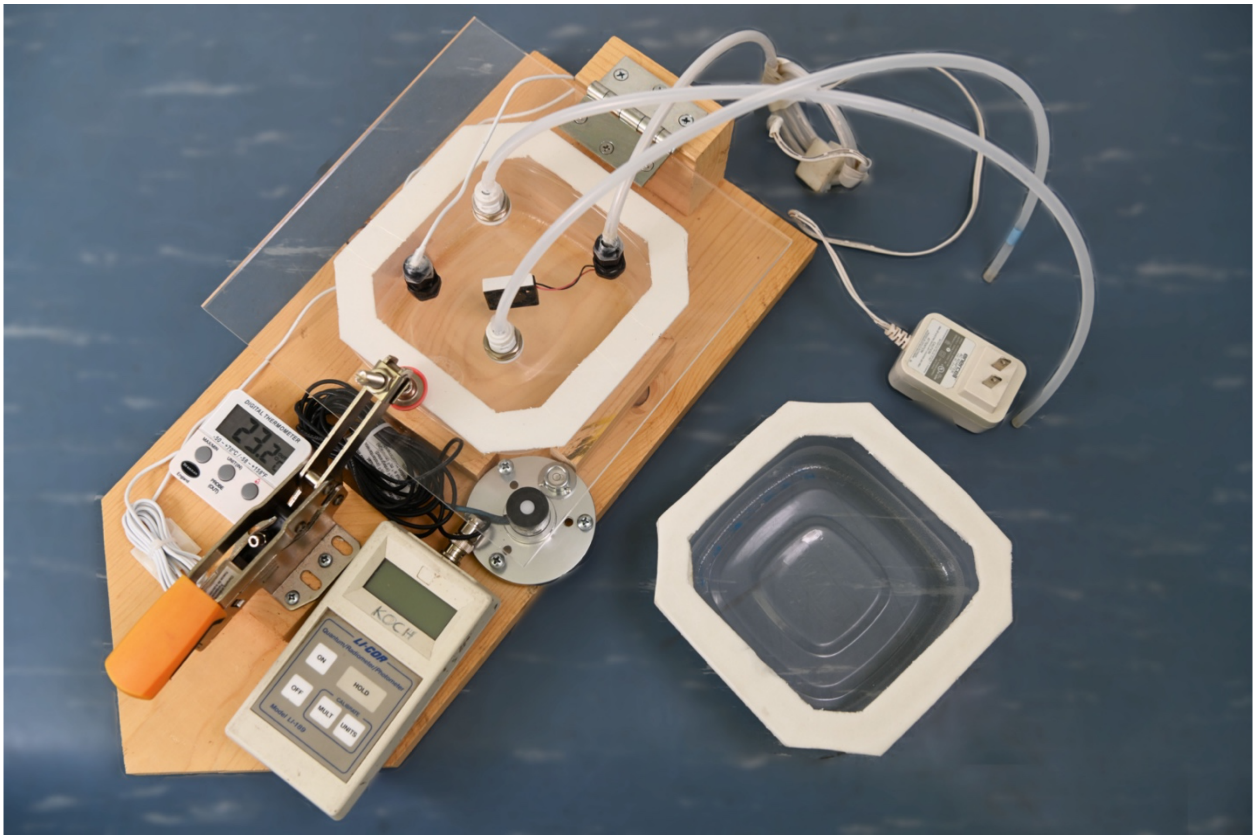

Supplementary Figure 1. Setup of the chamber system used to measure trace gas fluxes, and the temperature sensor and quantum sensor used for environmental measurements.

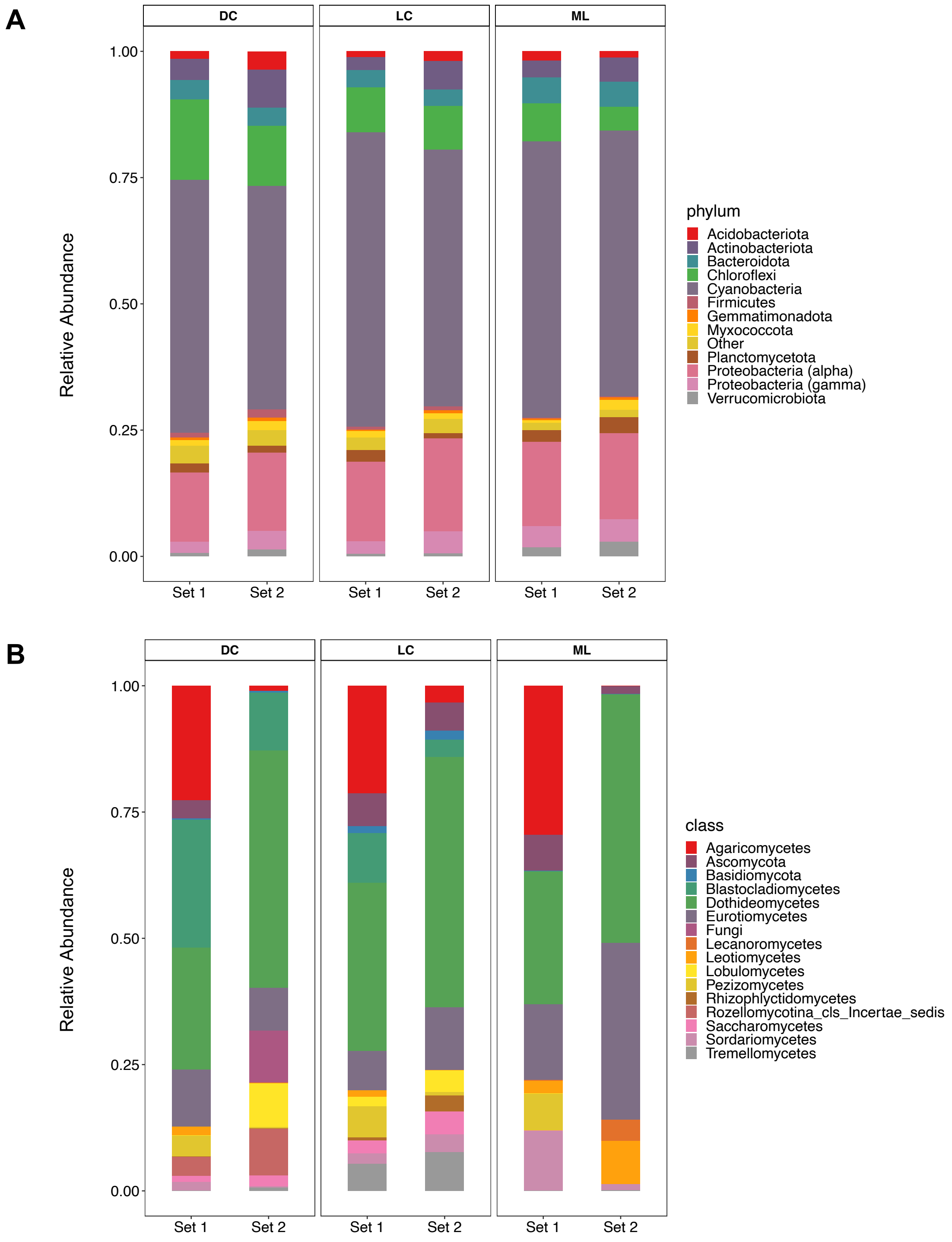

Supplementary Figure 2: Bar plots depicting average community composition (relative abundance) in each biocrust type for (A) bacteria (at phylum level) and (B) fungi (at class level). Results are broken down by samples from inoculation set 1 and inoculation set 2. Set 1 samples were about 8 weeks older (more developed) than set 2 samples. Sequencing was conducted on subsamples from two of the 10 samples of each biocrust type and set.

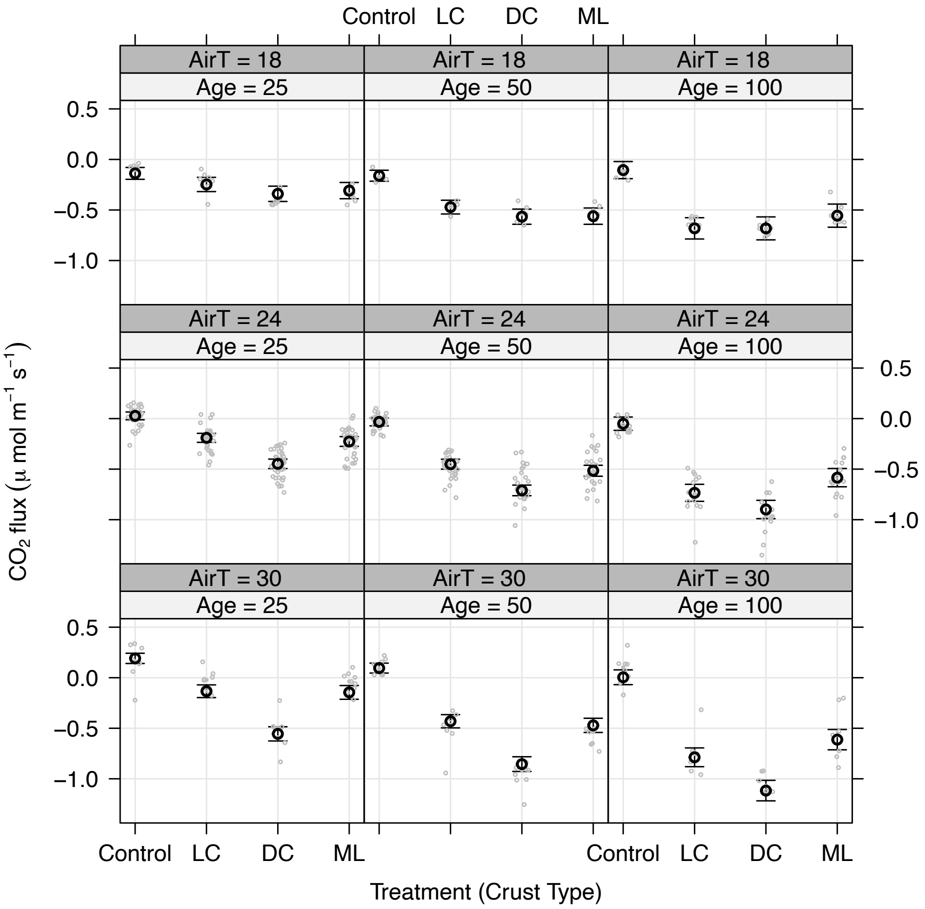

Supplementary Figure 3. Treatment effect plots showing differences among treatments (biocrust types; control, LC: light cyanobacteria, DC: dark cyanobacteria, ML: moss-lichen) in CO_2 flux rates at ages (Age, days) of 25, 50, and 100 days since inoculation, and temperatures (AirT, °C) of 18, 24, and 30°C, based on mixed effects model analysis of weekly flux measurements. Negative fluxes denote CO_2 uptake by the biocrusts. Open symbols indicate estimated effects, error bars are 95% confidence intervals for the effects, and grey dots are partial residuals of the effect.

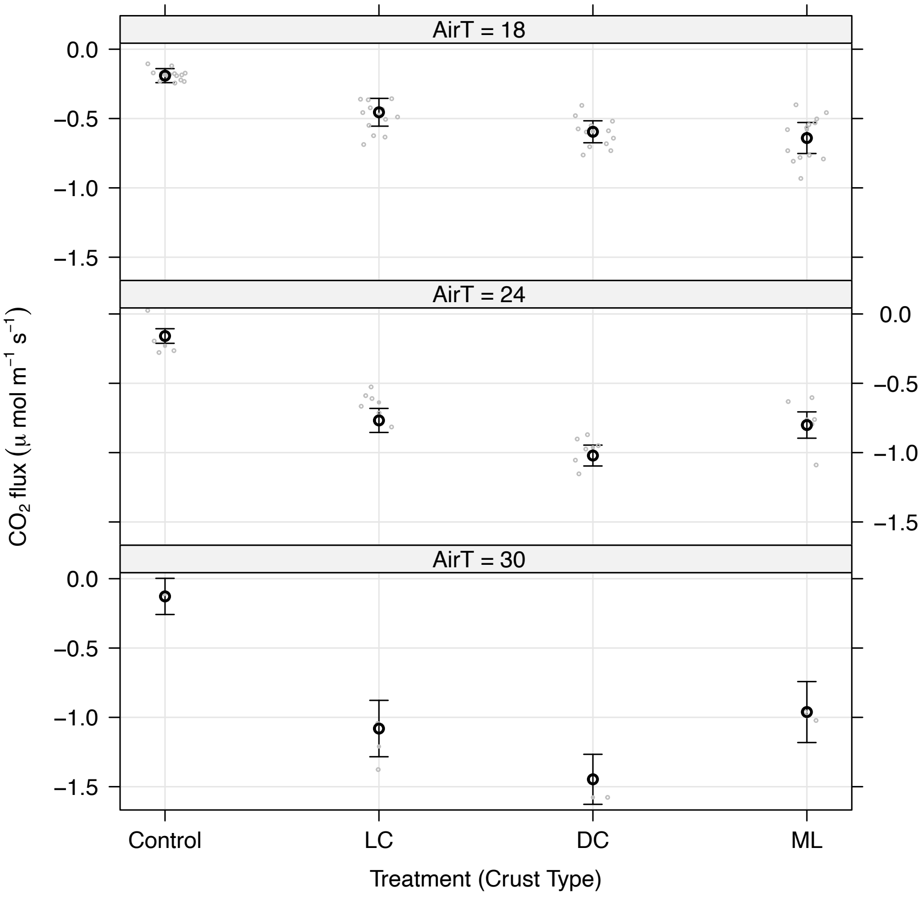

Supplementary Figure 4. Treatment effect plots showing differences among treatments (biocrust types; control, LC: light cyanobacteria, DC: dark cyanobacteria, ML: moss-lichen) in CO_2 flux rates at ambient air temperatures (AirT, °C) from 18°C to 30°C, based on mixed effects model analysis of end-of-summer flux measurements, when biocrusts. Negative fluxes denote CO_2 uptake by the biocrusts. Open symbols indicate estimated effects, error bars are 95% confidence intervals for the effects, and grey dots are partial residuals of the effect.

1.2 Supplementary Tables

Supplementary Table 1. Type III (partial) output of the mixed effects model for weekly CO_2 flux measurements (n = 473). For F-tests NumDF is numerator degrees of freedom; there were 293 denominator degrees of freedom for all effects except Treatment, which had 156 denominator degrees of freedom. Effects are: Treatment = soil biocrust type (control, light cyanobacteria, dark cyanobacteria, moss-lichen), Age = biocrust age in days since inoculation, AirT = ambient air temperature (°C) at the time of measurement, Light = incident solar radiation (PPFD, µmol m^{-2} s^{-1}) at the time of measurement, SoilMoist = gravimetric soil moisture (g H_2O/g oven dry soil) at the time of measurement.

**Effect**	**NumDF**	***F*-value**	***p*-value**
(Intercept)	1	49.52	<0.0001
Treatment	3	18.25	<0.0001
Age	1	0.63	0.4263
I(Age^2)	1	4.09	0.0439
AirT	1	89.61	<0.0001
Light	1	14.77	0.0001
I(Light^2)	1	12.68	0.0004
SoilMoist	1	32.97	<0.0001
Age:AirT	1	20.11	<0.0001
AirT:SoilMoist	1	46.46	<0.0001
Treatment:Age	3	19.18	<0.0001
Treatment:I(Age^2)	3	6.96	0.0002
Treatment:AirT	3	32.16	<0.0001

Supplementary Table 2. Type III (partial) output of the ANCOVA model for weekly CH_4 flux measurements (n = 468). For F-tests NumDF is numerator degrees of freedom; there were 451 denominator degrees of freedom. Effects are: Treatment = biocrust type (control, light cyanobacteria, dark cyanobacteria, moss-lichen), Age = biocrust age in days since inoculation, AirT = ambient air temperature (°C) at the time of measurement, Light = incident solar radiation (PPFD, µmol m^{-2} s^{-1}) at the time of measurement, SoilMoist = gravimetric soil moisture (g H_2O/g oven dry soil) at the time of measurement.

**Effect**	**NumDf**	***F*-value**	***p*-value**
(Intercept)	1	27.47	<0.0001
Treatment	3	6.48	0.0003
Age	1	33.44	<0.0001
Days	1	34.15	<0.0001
I(Days^2)	1	7.69	0.0058
Light	1	8.31	0.0041
I(Light^2)	1	4.88	0.0276
SoilMoist	1	8.60	0.0035
AirT	1	31.40	<0.0001
I(AirT^2)	1	33.34	<0.0001
I(Days*AirT)	1	36.02	<0.0001

Supplementary Table 3. Type III (partial) output of the ANCOVA model for end-of-summer CO_2 flux measurements (n = 79). For F-tests, NumDF is numerator degrees of freedom, and there were 71 denominator degrees of freedom. Effects are: Treatment = biocrust type (control, light cyanobacteria, dark cyanobacteria, moss-lichen) and AirT = ambient air temperature (°C) at the time of measurement.

**Effect**	**NumDF**	***F*-value**	***p*-value**
(Intercept)	1	3.74	0.0569
Treatment	3	6.01	0.0010
AirT	1	0.56	0.4563
Treatment:AirT	3	15.43	<0.0001

Supplementary Table 4. Type III (partial) output of the ANCOVA model for the end-of-summer CH_4 flux measurements (n = 79). For F-tests, NumDF is numerator degrees of freedom, and there were 74 denominator degrees of freedom. Effects are: Treatment = biocrust type (control, light cyanobacteria, dark cyanobacteria, moss-lichen) and AirT = ambient air temperature (°C) at the time of measurement.

**Effect**	**numDF**	***F*-value**	***p*-value**
(Intercept)	1	23.81	<0.0001
Treatment	3	0.74	0.5292
AirT	1	128.81	<0.0001

Supplementary Table 5. Type III (partial) output of the ANCOVA model for the end-of-summer N_2O flux measurements (n = 80). For F-tests: NumDF is numerator degrees of freedom, and there were 74 denominator degrees of freedom. Effects are: Treatment = biocrust type (control, light cyanobacteria, dark cyanobacteria, moss-lichen) and Light = incident solar radiation (PPFD, µmol m^{-2} s^{-1}) at the time of measurement.

**Effect**	**numDF**	***F*-value**	***p*-value**
(Intercept)	1	10.81	0.0015
Treatment	3	2.36	0.0786
Light	1	10.65	0.0017
I(Light^2)	1	6.90	0.0105
